# Fault Detection of Cyber-Physical Systems Using a Transfer Learning Method Based on Pre-Trained Transformers

**DOI:** 10.3390/s25134164

**Published:** 2025-07-04

**Authors:** Pooya Sajjadi, Fateme Dinmohammadi, Mahmood Shafiee

**Affiliations:** 1 School of Computing and Engineering, University of West London, London W5 5RF, UK; 2 School of Engineering, University of Surrey, Guildford GU2 7XH, UK; m.shafiee@surrey.ac.uk

**Keywords:** cyber-physical systems (CPSs), prognostics and health management (PHM), machine learning (ML), fault detection and diagnosis, transformers, transfer learning, explainable artificial intelligence (XAI)

## Abstract

**Highlights:**

**What are the main findings?**

- A pre-trained transformer model, fine-tuned with transfer learning, significantly improves fault detection in cyber-physical systems (CPSs) despite limited fault-labeled data.
- The proposed method achieves a high average F1-score of 93.38% on industrial CPS datasets, outperforming traditional CNN and LSTM models.

**What is the implication of the main finding?**

- Transformer-based transfer learning enables more reliable fault diagnostics in industrial CPS environments where data scarcity and domain shifts are common.
- The approach demonstrates practical scalability from controlled lab conditions to real-world industrial applications.

**Abstract:**

As industries become increasingly dependent on cyber-physical systems (CPSs), failures within these systems can cause significant operational disruptions, underscoring the critical need for effective Prognostics and Health Management (PHM). The large volume of data generated by CPSs has made deep learning (DL) methods an attractive solution; however, imbalanced datasets and the limited availability of fault-labeled data continue to hinder their effective deployment in real-world applications. To address these challenges, this paper proposes a transfer learning approach using a pre-trained transformer architecture to enhance fault detection performance in CPSs. A streamlined transformer model is first pre-trained on a large-scale source dataset and then fine-tuned end-to-end on a smaller dataset with a differing data distribution. This approach enables the transfer of diagnostic knowledge from controlled laboratory environments to real-world operational settings, effectively addressing the domain shift challenge commonly encountered in industrial CPSs. To evaluate the effectiveness of the proposed method, extensive experiments are conducted on publicly available datasets generated from a laboratory-scale replica of a modern industrial water purification facility. The results show that the model achieves an average F1-score of 93.38% under K-fold cross-validation, outperforming baseline models such as CNN and LSTM architectures, and demonstrating the practicality of applying transformer-based transfer learning in industrial settings with limited fault data. To enhance transparency and better understand the model’s decision process, SHAP is applied for explainable AI (XAI).

## 1. Introduction

As technology continues to evolve, there is a growing trend toward integrating the Internet to enhance the connectivity of devices and systems, particularly with the increasing use of Internet of Things (IoTs) devices. This development has led to the embedding of cyber components within physical systems, enabling real-time monitoring and control of physical processes [1]. Such integration, known as cyber-physical systems (CPSs), significantly improves the efficiency and controllability of these systems. CPSs are increasingly being deployed across critical industries, including transportation [2], healthcare [3], energy [4], and manufacturing [5]. However, the tight integration of cyber and physical components also introduces potential vulnerabilities, such as increased susceptibility to cyberattacks, sensor failures, and software malfunctions.

As industries seek more flexible, efficient, and reliable systems, ensuring the resilience of CPS is essential to prevent cascading failures and minimize economic losses. To address these challenges, the industry is increasingly adopting Prognostics and Health Management (PHM) as a strategic approach to enhance system reliability, improve operational efficiency, and support the long-term sustainability of complex systems [6]. PHM serves as a comprehensive computational framework that combines physical modeling, real-time operational data, and domain-specific expertise to assess the health of system components. By continuously monitoring system performance and detecting early signs of degradation, PHM enables the forecasting of potential failures and supports data-driven maintenance planning. This proactive approach not only reduces unplanned downtime and maintenance costs but also enhances overall system reliability, safety, and lifecycle performance.

**Figure 1:**
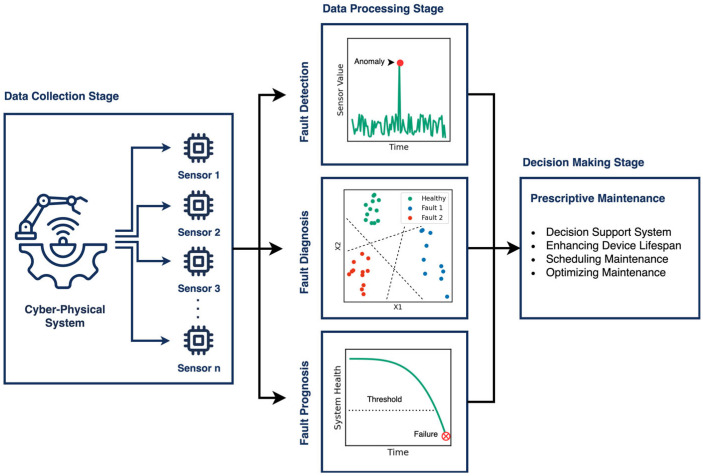
A typical flow diagram of a PHM framework for CPSs.

Figure 1 presents a typical flow diagram of a PHM framework for CPS. The process begins with data acquisition, where sensors collect operational data from the system. This raw data is then processed to support objectives such as fault detection, diagnosis, or prognosis, depending on the system’s specific requirements and the availability of data. Fault detection involves identifying anomalies or failures, whereas fault diagnosis extends this process by localizing, classifying, and assessing the nature and severity of the detected faults. Fault prognosis, on the other hand, aims to predict the timing and location of potential failures, often by estimating the system’s future condition or the remaining useful life (RUL) of its components [7,8,9]. Building on these insights, prescriptive maintenance offers a more advanced, data-driven approach by recommending targeted actions to maintain or restore optimal system performance. It goes beyond prediction to support informed decision-making, enhance operational efficiency, and drive greater business value through strategic maintenance planning [10,11].

Advancements in the IoT have greatly increased the availability of data from various components of CPS. This abundance of data offers a valuable opportunity to develop advanced data-driven models capable of extracting critical insights into system performance and health conditions. However, despite this data availability, several challenges continue to hinder the widespread adoption of data-driven methodologies in industrial settings. One of the primary challenges is the complexity of data pre-processing and manual feature extraction, which are both labor-intensive and technically demanding tasks [12]. Due to the heterogeneous nature of CPS components, feature extraction must be specifically tailored to the unique characteristics, operational conditions, and functional roles of each component, further complicating the process [13,14].

In recent years, deep learning (DL) techniques have shown great promise in addressing data analysis challenges, owing to their ability to autonomously learn and extract complex patterns from raw, unprocessed data [15,16]. However, DL models are inherently data-dependent and require large, diverse datasets to produce accurate and reliable diagnostics of system health states. While modern industrial environments generate substantial volumes of data, a major challenge arises from the dynamic and evolving operating conditions of CPS. Data collection under abnormal or fault conditions is often limited, leading to highly imbalanced datasets that hinder model performance [17]. Furthermore, the operational profiles of CPS frequently shift over time, making it both difficult and costly to obtain and label representative fault data, especially in environments where safety concerns and economic constraints severely limit fault data acquisition.

To address the challenges outlined above, this paper proposes a novel approach employing transfer learning with pre-trained transformers for fault detection in CPS. Originally introduced by Vaswani et al. [18], transformers are powerful, parallelized models designed for processing sequential data. They have demonstrated remarkable success across various domains, including natural language processing (NLP) and image recognition. Given the sequential nature of sensor data in CPS, transformers are particularly well-suited for this application. Unlike Recurrent Neural Networks (RNNs), which process data in a recurrent manner, transformers use attention mechanisms to capture dependencies across entire sequences more efficiently and in parallel [19]. The attention mechanism enables the model to assign contextual importance to different elements within a sequence, regardless of their position, thereby enhancing its ability to extract meaningful patterns. However, transformers are typically large models that require extensive training data to perform effectively. To overcome this limitation, we apply transfer learning, a technique that utilizes knowledge acquired from a data-rich source domain to improve performance on a related, data-scarce target domain. This approach enables better generalization by transferring learned features, making it especially valuable in DL contexts where labeled data is limited [20].

In this study, we first pre-train a transformer model on a large, labeled dataset collected from a controlled laboratory environment. This initial training phase allows the model to learn robust feature representations. We then fine-tune the pre-trained model on a smaller dataset obtained under different operating conditions, enabling the model to adapt and generalize to previously unseen fault scenarios, even with limited data availability. To the best of our knowledge, this work is among the first to develop a pre-trained transformer architecture combined with transfer learning for fault detection in CPS. The proposed models will be evaluated using the Secure Water Treatment (SWaT) dataset, which is derived from a scaled-down industrial water treatment testbed [21]. Developed by the iTrust Centre for Research in Cyber Security at the Singapore University of Technology and Design, the dataset contains time-stamped data capturing both normal operations and a variety of cyber-physical attack scenarios. It includes sensor readings, actuator states, and network traffic, providing a comprehensive representation of real-world CPS behavior.

While prior research in PHM has explored transformers and transfer learning independently, our study focuses on the underexplored problem of generalizing PHM models under domain shift, which is a common issue in CPS. Our approach is based on using a transformer model that is first pre-trained on a comprehensive laboratory dataset and then fine-tuned on a smaller dataset collected under different operating conditions. This method addresses the mismatch between controlled laboratory data and real-world environments, which has received limited attention in the existing literature.

Although pre-training and fine-tuning transformer models are well-established in general AI, their specific use for fault detection under domain shift in CPS has not been studied in depth. In addition, many existing approaches assume access to large, balanced datasets or do not consider model interpretability. These assumptions limit their practical use in industrial PHM settings where fault data are often scarce. To address these issues, we employ a simplified transformer architecture, a domain adaptation strategy suitable for small datasets, and model explainability through SHapley Additive exPlanations (SHAP) [22], which aids in interpreting model decisions.

Deep learning models are often seen as black boxes, which can be a concern in safety-critical applications such as CPS fault detection. To improve transparency, we apply the SHAP method to analyze the contribution of input features to model predictions and to support system-level decision-making. The main contributions of this paper are summarized as follows:

- We propose a transfer learning approach for PHM of CPS by using pre-trained transformer models to address significant discrepancies between source and target datasets, particularly in terms of operating conditions.
- An end-to-end fine-tuning strategy is employed, incorporating cross-validation under severe class imbalance and a simplified architecture using a single encoder-decoder transformer. This design makes transfer learning feasible for small PHM datasets, which is an aspect often overlooked in previous transformer-based PHM studies.
- The proposed methodology enables effective knowledge transfer from laboratory-controlled datasets to real-world operational data, achieving improved fault detection performance as validated through extensive experiments on the SWaT dataset, outperforming existing DL-based approaches.
- To increase model interpretability and support decision-making, the SHAP method is used on the fine-tuned transformer model, which provides insight into the model’s internal behavior and improves transparency in fault diagnosis.

The remainder of the paper is organized as follows. Section 2 reviews related work and the current state of the art. Section 3 describes the proposed methodology in detail. Section 4 outlines the experimental setup and procedures. Section 5 presents the results and discusses their implications. Finally, Section 6 concludes the study and highlights potential directions for future research.

## 2. Literature Review

In recent years, there has been increasing interest in applying DL techniques to the PHM of CPS. This shift is driven by the limitations of traditional machine learning models in handling the complex, high-dimensional sensor data produced by these systems. DL-based approaches are primarily aimed at monitoring system performance to enable fault detection, diagnosis, and prognosis. The ultimate goal is to support intelligent maintenance scheduling and reduce unexpected disruptions by using the rich sensor data generated during system operation.

However, despite progress, the application of DL in PHM, particularly in real-world, data-constrained environments, faces ongoing challenges, including data imbalance, lack of interpretability, and limited generalizability under domain shifts. This section reviews the evolution of DL in PHM, categorized into feedforward/convolutional models, temporal models, and recent transformer-based and transfer learning approaches, while focusing on current limitations and the unique contributions of this study.

### 2.1. Feedforward and CNN-Based Approaches

Early applications of DL in PHM primarily utilized fully connected (FC) networks and Convolutional Neural Networks (CNNs) to classify fault states or estimate remaining useful life (RUL). Yang et al. [23] applied a 1D CNN model on the IEEE PHM Challenge 2012 dataset for both fault classification and RUL estimation from vibration signals, demonstrating superior performance over Support Vector Regression (SVR). Similarly, Lupea et al. [24] used 2D CNNs with Continuous Wavelet Transform (CWT) for defect classification in gearboxes, achieving over 99% accuracy across multiple failure types.

Several studies have sought to enhance CNN-based methods through architectural innovations or hybrid designs. For example, Wei et al. [25] combined residual networks (ResNets) with Extreme Learning Machines (ELMs) to create a model capable of extracting robust time-frequency features for fault classification in rotating machinery. Cui et al. [26] introduced generative modeling through a Conditional Wasserstein Generative Adversarial Network with Gradient Penalty (CWGAN-GP) to augment imbalanced fault datasets and improve classifier performance using a dual-CNN architecture. Despite their effectiveness, these CNN-based methods typically treat time as a static dimension and are thus limited in their ability to capture temporal dependencies, a critical aspect of sensor data in dynamic systems.

### 2.2. Sequential Models and Hybrid Architectures

To overcome the limitations of static models, research attention has increasingly shifted toward sequential models that can account for time-dependent behavior in CPS data. Long Short-Term Memory (LSTM)-based models have shown particular promise in this area. Bampoula et al. [27] developed an LSTM autoencoder framework to monitor equipment health over time, using temperature and hydraulic sensor data from milling machines. Shoorkand et al. [28] proposed a hybrid CNN–LSTM–FC model that integrated convolutional feature extraction with LSTM-based temporal modeling to improve predictive maintenance accuracy.

Hybrid architectures are increasingly favored in PHM for their ability to combine spatial and temporal learning. Wang et al. [29] proposed a model combining 1D CNN, Bi-LSTM, and attention layers to predict RUL from aircraft engine data. The fusion of local convolutional features and long-term dependencies allowed for better prediction of component degradation over time. Likewise, Luo et al. [30] applied a 1D CNN–BiLSTM–Attention model to vibration data from bearings and showed improved early fault prediction accuracy, though they noted difficulties with capturing early-stage degradation.

While these models address the issue of temporal dependencies, they often suffer from long training times, sensitivity to sequence length, and challenges in learning long-range relationships, especially in large-scale multivariate time series data.

### 2.3. Transformer-Based Models for PHM

Transformers, originally introduced by Vaswani et al. [18], have reshaped sequence modeling by using attention mechanisms to capture long-range dependencies without the need for recurrence. Unlike LSTMs, transformers process input sequences in parallel, making them more computationally efficient and better suited for modern sensor streams. Their growing popularity in time-series domains has led to their recent application in PHM tasks.

Zhang et al. [19] introduced a Dual-Aspect Self-Attention Transformer (DAST) model for RUL prediction, using sliding windows over raw sensor data to extract temporal features through attention layers. Chen et al. [31] proposed an attention-based LSTM model, combining manually engineered features with deep features. While the proposed method outperformed existing models in terms of prediction accuracy, it exhibited limitations when applied to varying operational conditions.

These approaches demonstrated that attention-based architectures could outperform traditional RNN and CNN models in both accuracy and generalization. However, transformers typically require large volumes of labeled data for effective training. In industrial CPS environments, fault data are scarce, costly to collect, and often imbalanced. As such, the direct application of transformer models without any adaptation remains limited in real-world PHM systems.

### 2.4. Transfer Learning in PHM

While CPS generates vast quantities of data, acquiring labeled data across the full range of operating conditions remains a significant challenge, particularly in industrial environments where systems are designed to operate reliably with minimal faults. As a result, collecting comprehensive labeled datasets is often impractical. To overcome this limitation, transfer learning can be employed. This approach involves training models on extensive datasets gathered in controlled laboratory settings and then transferring the learned knowledge to real-world industrial environments, thereby reducing the reliance on large-scale data collection under operational conditions [32].

To date, only a limited number of studies have explored transfer learning as a viable approach for deploying deep and complex architectures in data-constrained environments. For example, Berghout et al. [33] proposed a DL model based on an LSTM-FC architecture, incorporating transfer learning by pre-training the LSTM layers on a subset of the PHM 2012 Challenge bearing dataset. The model was then fine-tuned on a different subset of the same dataset, with the LSTM weights kept frozen during the fine-tuning process. In another study, Sanakkayala et al. [34] transformed bearing vibration signals into spectrograms to generate 2D image representations and used pre-trained DL models such as ResNet-50 for feature extraction. The extracted features were subsequently passed to fully connected layers for fault classification.

Despite these promising efforts, the application of transfer learning for fault detection in CPS remains limited, particularly in the context of using pre-trained transformer models. This gap presents a valuable research opportunity, as integrating transfer learning with transformer architectures holds significant potential to enhance fault detection performance while addressing the persistent challenge of data scarcity in real-world industrial environments.

To address these gaps, this study presents a transfer learning approach based on a pre-trained transformer architecture, which is fine-tuned on a smaller target dataset with differing operating conditions. In addition, SHAP model explainability is incorporated to support interpretation of the model’s predictions. Together, these elements aim to improve the applicability of DL models for fault detection in real-world CPS settings where labeled fault data is limited.

## 3. Methodology

**Figure 2:**
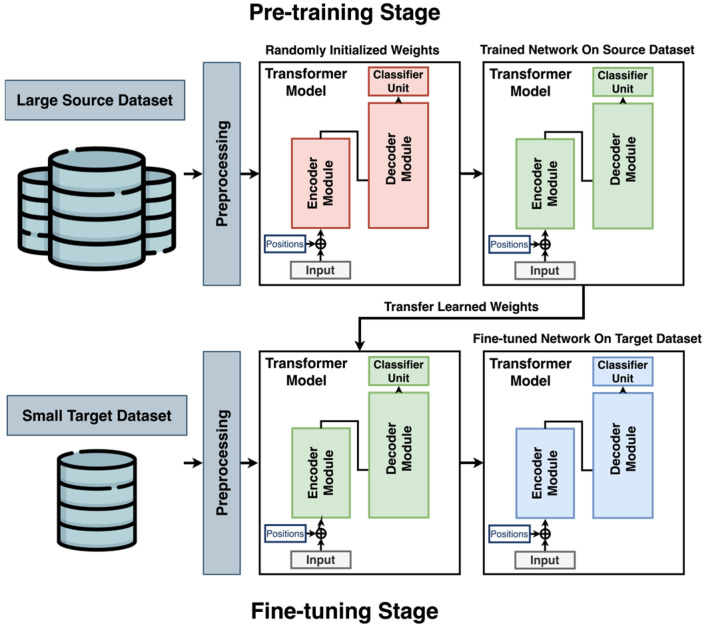
The proposed transfer learning approach.

Figure 2 illustrates the proposed transfer learning framework, which utilizes a transformer-based architecture to enable effective knowledge transfer from a large-scale source domain to a smaller, domain-specific target domain. In this study, the DL model is initially pre-trained on a large-scale source dataset until it achieves optimal performance. Subsequently, the model is fine-tuned and validated on the target dataset using K-fold cross-validation to mitigate overfitting and reduce bias. As depicted, the model begins with randomly initialized weights (indicated in red), which are updated during the pre-training phase. These trained weights (shown in green) serve as the starting point for the fine-tuning stage, during which they are further adjusted until convergence (shown in blue). This process enables the model to use knowledge from the source domain to improve performance on the target dataset, which contains limited labeled data. The transformer architecture is chosen for its strong ability to capture temporal dependencies and detect patterns in sequential data.

**Figure 3:**
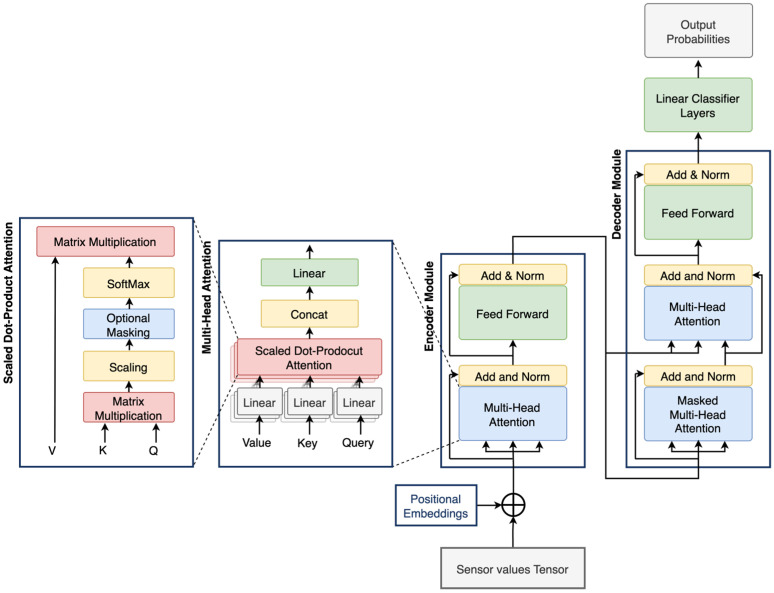
Transformer architecture used in the study.

The proposed framework differs from existing approaches in two key aspects. First, it introduces a simplified transformer architecture (shown in Figure 3) specifically designed to accommodate the constraints of small-scale PHM datasets, in contrast to traditional transformer models developed for large-scale applications. Second, it incorporates a transfer learning strategy to improve model adaptability in the presence of domain shift, a common challenge in real-world CPS scenarios. The following sections provide a detailed explanation of the architectural design and training methodology.

### 3.1. Transformer Architecture

Transformers are advanced DL architectures designed to extract meaningful insights from sequential data while accounting for positional context. As illustrated in Figure 3, a typical transformer consists of several components, such as positional encoding layers, encoder and decoder stacks, and attention mechanisms [18]. In this study, the transformer architecture has been simplified to suit the scale and complexity of the available PHM datasets. Unlike conventional transformer models that employ deep encoder and decoder stacks, the proposed architecture utilizes a single encoder and a single decoder module. This design choice aims to reduce model complexity while retaining the fundamental strengths of the transformer architecture.

The model begins with a positional encoding layer that injects time-related information into the input vectors. This is followed by multi-head attention mechanisms within both the encoder and decoder, enabling the model to learn diverse dependencies in parallel. Finally, a stack of fully connected layers performs the classification task. By adopting this framework, the model achieves strong performance with limited target-domain data, reducing the risk of overfitting and improving suitability for deployment in resource-constrained industrial settings.

#### 3.1.1. Positional Encoding Layer

Unlike recurrent and convolutional models, transformers do not inherently capture the sequential order of input data. To address this limitation, a positional encoding layer is incorporated to embed positional information into the input vectors. These encodings, which have the same dimensionality as the model inputs, are added directly to the input vectors. Although positional encodings can be defined as trainable parameters, we adopt fixed sinusoidal functions with varying frequencies as introduced in [18] to reduce model complexity. The corresponding formulas for these encodings are presented in Equations (1) and (2), where POS denotes the position index and i refers to the dimension.

(1) $${PE}_{\left(POS,2i\right)}=sin\;(pos/1000^{2i/d_{model}})$$

(2) $${PE}_{\left(POS,2i+1\right)}=cos\;(pos/1000^{2i/d_{model}})$$

#### 3.1.2. Attention Layers

At the core of the transformer model is the attention mechanism, which enables the model to identify meaningful patterns in sequential sensor data [35]. Specifically, the attention layer computes three distinct, learned representations of the input tensor via linear transformations: the query (Q), which specifies what we are searching for; the key (K), which indicates what information is available; and the value (V), which contains the content to be retrieved. These projections are mathematically defined in Equation (3):

(3) $$Q=W_{q}\times X,\;\;K={\;W}_{k}\times X,\;\;V={\;W}_{V}\times X$$

In this study, we utilize scaled dot-product attention to compute the attention weights (α), as shown in Equation (4). In this formulation, the query, key, and value vectors all share the same dimensionality, denoted by $d_{q}=d_{k}=d_{v}$. The attention score is calculated by taking the dot product of the query and key vectors, which measures their similarity. This score is then scaled by the square root of the model dimension $D_{model}$ to stabilize gradients and passed through a softmax function to produce a probability distribution over the input sequence:

(4) $$\alpha \left(Q,K\right)=Softmax(\frac{QK^{T}}{\sqrt{D_{model}}})$$

The softmax-weighted distribution determines the degree of attention allocated to each element in the input sequence. The output of the attention mechanism is then computed by multiplying these attention scores with the corresponding value vectors, as shown in Equation (5).

(5) $$Attention\left(Q,K,V\right)=\;\alpha (Q,K)V\;$$

Among various attention mechanisms, scaled dot-product attention has been widely adopted due to its balance of computational efficiency and empirical effectiveness [18]. Finally, in the case of the multi-head attention mechanism, multiple attention heads are independently trained, and their outputs are concatenated to form the final representation, as shown in Equation (6) with X being the input sequence, h representing the number of heads, ${Attention}_{i}$ being the calculated attention vector for each individual head, and $W^{o}$ as the output projection matrix to combine the heads.

(6) $$MHAttention\left(X\right)=\;Concat({Attention}_{1},\;\ldots ,{Attention}_{h})\;W^{o}$$

#### 3.1.3. Encoder Stack

The encoder stack consists of multiple sublayers, each comprising a multi-head attention mechanism followed by an FC feed-forward layer. Both components are wrapped with residual connections, layer normalization, and dropout layers to enhance training stability. Each encoder layer maps the input tensor to a fixed-dimensional representation of size $D_{model}$. The use of residual connections helps mitigate the vanishing gradient problem, facilitating the training of deep networks. Furthermore, the multi-head attention mechanism enables the model to simultaneously focus on different parts of the input by computing multiple attention distributions in parallel. This parallelism allows the model to capture diverse relationships and patterns within the sequential sensor data, which is essential for effective fault detection in CPS. The encoder stack is defined in Equations (7) and (8).

(7) $$Z=LN\left(X+Dropout\left(MHAttention\left(X\right)\right)\right)$$

In Equation (7), Z represents the output of the first add and normalize sub-layer, $LN\left(.\right)$ denotes the layer normalization function, and FFN(.) is the feed-forward network.

(8) $${Output}_{Encoder}=LN\left(Z+Dropout\left(FFN\left(Z\right)\right)\right)$$

#### 3.1.4. Decoder Stack

Similar to the encoder stack, the decoder stack in a transformer model consists of multiple identical layers, each comprising three subcomponents: two multi-head attention mechanisms and a fully connected feed-forward network. Each sublayer is enclosed in a residual connection followed by layer normalization, which enhances training stability and supports better gradient flow. The first attention layer in the decoder is typically a self-attention mechanism. The second is a cross-attention mechanism with Query taken from the decoder and key and value from the encoder, which enables the decoder to attend to the encoder’s output. This enables the integration of contextual information from the input sequence, improving the model’s ability to generate accurate and context-aware predictions. Overall, the decoder is shown in Equations (9)–(11).

(9) $$Z_{1}=LN\left({Output}_{Encoder}+Dropout\left(MHAttention({Output}_{Encoder}\right)\right)$$

In Equation (9), $Z_{1}$ is the self-attention sub-layer output using directly the output from the encoder. In Equation (10), $Z_{2}$ is the cross-attention output.

(10) $$Z_{2}=LN\left(Z_{1}+Dropout\left(CrossAttention(Z_{1},{Output}_{Encoder}\right)\right)$$

Finally, Equation (11) calculates the decoder output using a residual connection and the feedforward network.

(11) $${Output}_{Decoder}=LN\left(Z_{2}+Dropout\left(FFN\left(Z_{2}\right)\right)\right)$$

#### 3.1.5. Classification Output

In the final stage, the output tensors produced by the decoder are passed through a series of FC layers, interleaved with Rectified Linear Unit (ReLU) activation functions. The classification head receives input vectors of dimension $D_{model}$, and the final output layer produces a probability distribution over the target classes. Model training is performed using the cross-entropy loss function, which is optimized via the gradient descent algorithm.

### 3.2. Transfer Learning

Transformer models have shown strong performance across various domains; however, their effectiveness diminishes when applied to small-scale PHM datasets due to their large model size and substantial data requirements. A practical solution for using the capabilities of transformers in such data-constrained scenarios is transfer learning. This approach involves pre-training a model on a larger, related source dataset (SD) and then fine-tuning it on a smaller, domain-specific target dataset (TD), thereby facilitating knowledge transfer between domains [12].

In PHM applications, data scarcity is particularly pronounced for fault scenarios, which are rare, expensive to simulate, and difficult to annotate. Moreover, the dynamic and evolving nature of industrial environments introduces domain shifts, making data collection even more challenging. To address these issues and enhance model generalizability under domain shift, this study adopts a transfer learning strategy tailored for PHM tasks.

#### 3.2.1. Pre-Training on the Source Domain

In the pre-training phase, the transformer model is trained on a large-scale dataset collected under operational conditions different from those of the target domain (as illustrated in Figure 2). This approach assumes that transferable temporal patterns exist across domains and can be captured by the model. The model is initialized with random weights and trained in a supervised classification setting until performance on the validation set ceases to improve. The resulting pre-trained weights form the foundation for subsequent adaptation to the target domain.

#### 3.2.2. Fine-Tuning on the Target Domain

In this phase, several fine-tuning strategies can be employed. These include (1) updating all model parameters, (2) freezing certain layers while fine-tuning others, and (3) employing a gradual unfreezing strategy, where fine-tuning starts with the final layers and progressively unfreezes earlier layers. The latter approach is frequently adopted in literature as it helps balance knowledge retention from the source domain with effective adaptation to the target domain, thereby reducing the risk of catastrophic forgetting [36]. In this study, multiple strategies are evaluated to determine the most effective method for transferring knowledge under data-constrained conditions.

### 3.3. Model Explainability

As DL models are often regarded as black boxes, it is critically important to interpret their outputs accurately, particularly in scenarios where model predictions serve as the basis for decision-making about complex systems, such as in the PHM of CPS. In such applications, both interpretability and reliability are essential attributes. While simpler ML models, such as decision trees, are inherently interpretable and therefore sometimes favored despite lower accuracy, the increasing complexity of systems and the growing volume of sensor data necessitate the use of more sophisticated models [22]. In this context, XAI techniques play a vital role by increasing the interpretability of complex models and enabling their adoption in critical decision-making processes.

In this study, the SHAP methodology is used to interpret the output of the fine-tuned model on the target dataset. SHAP quantifies the contribution of each input feature to the model’s prediction by evaluating how the output changes when the feature is included versus excluded, averaging over all possible subsets of features. The resulting SHAP value assigned to each feature reflects its relative impact on the prediction, with positive values indicating a positive contribution and negative values indicating a negative influence. Owing to its model-agnostic nature, SHAP can be applied post hoc to any trained predictive model, thereby providing a flexible and consistent framework for model interpretability.

Two key mathematical formulations form the basis of the SHAP framework and are fundamental to understanding how feature attributions are computed. The first is the SHAP explanation model, which expresses the prediction as a sum of individual feature contributions, as shown in Equation (12).

(12) $$g\left(z^{\prime }\right)=\phi_{0}+\;\sum_{i=1}^{M}\phi_{i}z_{i}\prime$$

Here, $g\left(z^{\prime }\right)$ is the explanation model, M is the number of features, $z^{\prime }\;\in {\left\{0,1\right\}}^{M}$ is a binary vector indicating the presence (1) or absence (0) of each feature, and $\phi_{i}$ is the SHAP value represents the contribution of feature i. The term $\phi_{0}$ corresponds to the baseline output of the model when no features are present. This formulation ensures that the sum of the SHAP values approximates the original model’s prediction and forms the basis for local interpretability.

The second key formula is the SHAP value computation, derived from cooperative game theory shown in Equation (13).

(13) $$\phi_{i}\left(f,x\right)=\sum_{S\subseteq F\backslash \{i\}}\frac{\left|S\right|!\left(\left|F\right|-\left|S\right|-1\right)!}{\left|F\right|!}\left[f_{S\cup \{i\}}\left(x_{S\cup \{i\}}\right)-f_{S}\left(x_{s}\right)\right]$$

In this expression, F is the full set of features, and S is a subset of F that does not contain feature i. The model predictions $f_{S}\left(x_{s}\right)$ and $f_{S\cup \{i\}}\left(x_{S\cup \{i\}}\right)$ represent the output of the model with and without feature i, respectively. In the formula, $\left|.\right|$ operator calculates the size of each set and ! operator is the factorial operator. The weighting term, based on factorials, ensures a fair distribution of contributions by averaging over all possible permutations of feature inclusion. This formulation guarantees three key properties—local accuracy, missingness, and consistency—which make SHAP values a theoretically sound measure of feature importance.

## 4. Experiment

To evaluate the performance of the proposed methodology, we applied it to two publicly available, real-world CPS sensor datasets: SWaT.A1 and SWaT.A6. These datasets, developed by iTrust at the Centre for Research in Cyber Security, Singapore University of Technology and Design, consist of time-series data from a six-stage water treatment system. The SWaT testbed is a laboratory-scale replica of a modern industrial water purification facility, designed to support research on the security and reliability of CPS. It operates continuously at approximately five gallons per minute and comprises six stages: raw water intake, chemical dosing, membrane filtration, ultraviolet dechlorination, reverse osmosis, and final water handling. Each stage is instrumented with a range of sensors and actuators, managed by programmable logic controllers (PLCs) and monitored through a Supervisory Control and Data Acquisition (SCADA) system.

Communication within the system is structured across two network layers, with wired links facilitating data transmission and collection. The datasets include readings from 51 sensors and actuators, such as flow meters, water level indicators, motorized valves, pumps, and pressure sensors, sampled at one second intervals. As one of the most comprehensive CPS datasets available, the SWaT dataset has been widely adopted in academic research on anomaly detection and system monitoring [37,38,39]. The SWaT.A1 dataset, recorded in 2015, captures 11 days of system operation: 7 days under normal conditions and 4 days during which 41 distinct cyber-physical attacks were conducted, resulting in a total of 449,920 data records. In contrast, the SWaT.A6 dataset, recorded in 2019, offers a shorter 4 h session comprising 13,202 data points, with the first 3 h representing normal operation and the final hour, including 6 unique attack scenarios.

**Figure 4:**
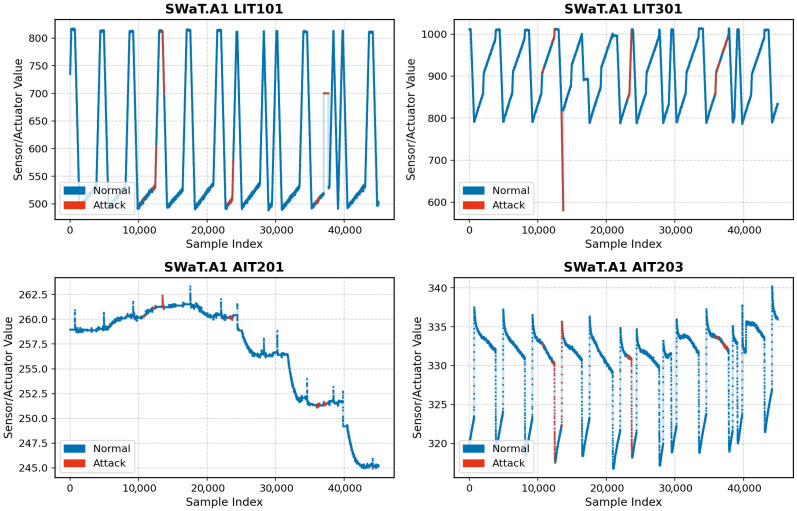
Four sampled sensor readings from the SWaT.A1 dataset under normal (blue) and attack (red) conditions.

Figure 4 illustrates sample sensor readings from the SWaT.A1 dataset, with normal and attack conditions highlighted in blue and red, respectively. While some attack events cause noticeable deviations in sensor signals that are easily identifiable, others result in more subtle variations, making them difficult to detect through visual inspection alone.

**Figure 5:**
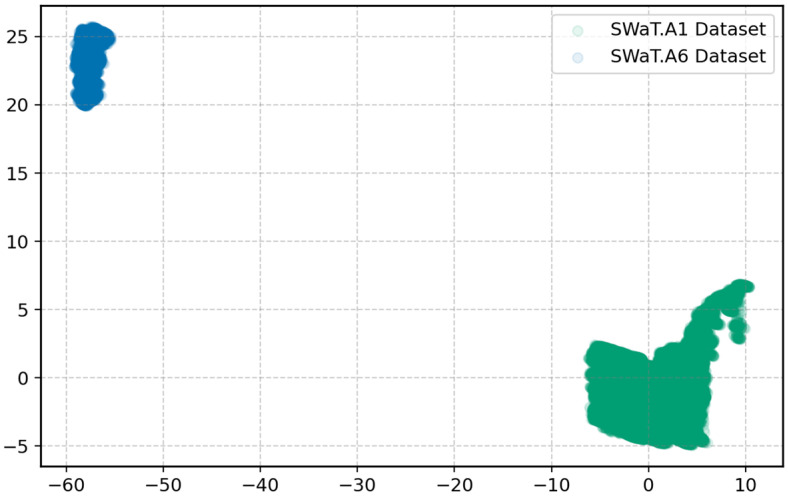
PCA representation of the normalized datasets.

Although both datasets originate from the same CPS system, they were collected under different operating conditions and recorded four years apart. Figure 5 shows a 2D visualization of the datasets using PCA after normalization. Each point represents a row in the respective datasets, forming two distinct clusters that highlight the variation in data distributions. The clear difference in probability densities shows the presence of a domain shift, which validates the suitability of a transfer learning approach. Given its limited size, the SWaT.A6 dataset alone is insufficient to train complex DL models such as transformer architectures. Therefore, this study uses a transfer learning strategy: the model is first pre-trained on the larger SWaT.A1 dataset and subsequently fine-tuned using the smaller SWaT.A6 dataset to enhance performance under data-constrained conditions.

The SWaT.A1 dataset is divided into training, validation, and test sets using a 70%, 15%, and 15% split, respectively. For pre-training, both normal and attack samples are used to perform a binary classification task, with the validation set used to monitor training progress and prevent overfitting. Once training convergence is achieved, the model is fine-tuned on the SWaT.A6 dataset. To assess the model’s repeatability and robustness, 10-fold cross-validation is applied. Initially, the SWaT.A6 dataset is split into 85% training and 15% testing subsets, with the test set held out for final evaluation. The training portion is further divided into 10 folds; in each iteration, 9 folds are used for training and 1 for validation. During training, model performance is tracked on the validation set, and the model checkpoint with the best validation score is selected for evaluation on the test set. Due to the significant class imbalance in the dataset, overall accuracy is not a suitable evaluation metric. Instead, the F1-score of the faulty (attack) class is prioritized, as it provides a more meaningful and fair comparison with state-of-the-art methods.

Feature selection was implicitly handled through the need for cross-dataset compatibility. Since SWaT.A1 and SWaT.A6 have overlapping but non-identical features, we retained only those common to both datasets to enable transfer learning

The preprocessing pipeline involves several steps: handling missing values, digitization, and normalization to enhance model robustness and accelerate convergence. Normalization is performed using standard score normalization (z-score), as shown in Equation (14), calculated based on the mean (μ) and standard deviation (σ) of the training set. This transformation is then applied uniformly across all data splits. Due to the sequential nature of the model, a sliding window technique is employed to segment the dataset into fixed-length subsequences.

(14) $$z=\frac{x-\mu \;}{\sigma }$$

Given the relatively small size of the SWaT datasets and the typically high capacity of transformer architectures, we adopt a simplified vanilla Transformer configuration consisting of a single encoder and a single decoder block. This lightweight setup is intentionally designed to balance model expressiveness with the risk of overfitting in low-data scenarios. By keeping the architecture compact, we ensure the model can still learn meaningful patterns while remaining robust and computationally efficient. Additionally, this design makes our method more practical for real-world settings, where labeled fault data is often scarce and large-scale pre-training is not always feasible. The specific model parameters used, summarized in Table 1, were selected through empirical testing and reflect the most promising trade-off between performance and generalizability.

All experiments were conducted using the Python programming language version 3.8.10, with the PyTorch library version 2.4.1 used for DL model construction, training, and validation. The experiments were executed on a GPU server equipped with an AMD Ryzen 3970X CPU with 64 cores, 128 GB of RAM, and an NVIDIA GeForce RTX 3090 GPU with 24 GB of memory. The equipment was sourced from the university’s facilities at the University of West London, London, UK.

## 5. Results and Discussions

In this section, we evaluate and discuss the performance of the proposed methodology using standard classification metrics. The following evaluation measures are employed to assess predictive accuracy: accuracy, precision, recall, and F1 Score, as defined in Equations (15)–(18):

(15) $$\mathrm{Accuracy}:\;\frac{TP+TN}{TP+TN+FP+FN}$$

(16) $$\mathrm{Precision}:\;\frac{TP}{TP+FP}$$

(17) $$\mathrm{Recall}:\;\frac{TP}{TP+FN}$$

(18) $$F1\;\mathrm{Score}:\;\frac{TP}{TP+\left(FP+FN\right)/2}$$

where TP denotes True Positives, TN denotes True Negatives, FP denotes False Positives, and FN denotes False Negatives. These metrics provide a robust assessment of classification performance, particularly in class-imbalanced scenarios, which are common in PHM tasks.

### 5.1. Pre-Training on SWaT.A1

**Figure 6:**
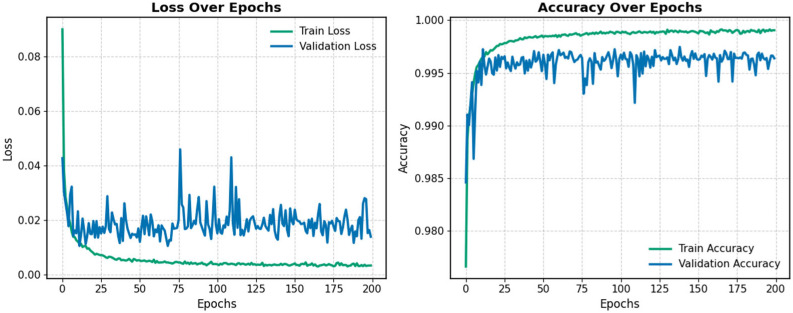
Training and validation chart during the pre-training stage.

In the first stage, the model was pre-trained on the SWaT.A1 dataset to extract useful information from the source domain. The training curves are shown in Figure 6. As illustrated, training was halted after 200 epochs due to signs of overfitting, indicated by stagnant validation performance. The model checkpoint with the highest validation accuracy was selected both for evaluating the test set and as the initialization point for fine-tuning on the target dataset.

Table 2 presents the experimental results and compares the proposed method with other state-of-the-art approaches on the test set of the SWaT.A1 dataset. Although improving performance on SWaT.A1 is not the primary goal of the methodology, the results show that the proposed model outperforms existing methods in terms of accuracy.

### 5.2. Fine-Tuning on SWaT.A6

**Figure 7:**
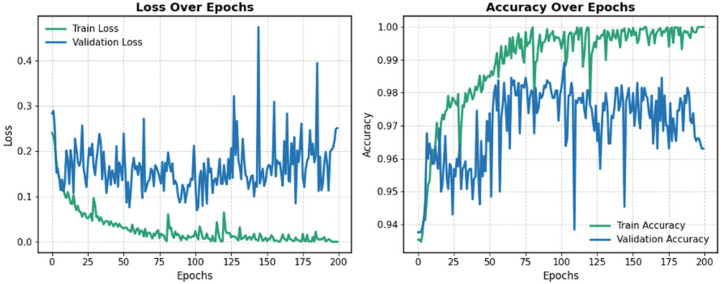
Training and validation chart during the fine-tuning stage.

Following pre-training on the SWaT.A1 dataset, the learned weights were used to fine-tune the model on the SWaT.A6 dataset. Figure 7 displays the training curves for loss and accuracy during the fine-tuning process for one of the folds. To mitigate overfitting and catastrophic forgetting, training progress was monitored using the validation set, employing the same strategy as in the pre-training phase. The model with the highest validation accuracy was selected and evaluated on the test set to determine final performance. Training was stopped after 200 epochs, as the loss curve had plateaued, and validation accuracy began to decline.

To evaluate the model’s performance, accuracy, F1-score, and *p*-values for F1-scores are reported in Table 3 and compared against state-of-the-art approaches using K-fold cross-validation. In addition to the proposed method, several variations were explored. To assess the impact of transfer learning, a baseline model with the same architecture was trained from scratch on the target dataset. Another variant involved freezing the transformer layers and fine-tuning only the classifier, which yielded lower performance compared to the proposed method.

As shown in Table 3, the proposed model outperforms commonly used baseline models reported in the literature. To ensure a fair and consistent evaluation, all baseline models were implemented in PyTorch based on architectural details reported in the literature. Each model was trained using the same normalized and preprocessed input data, cross-validation splits, and a sliding window approach with a fixed sequence length and input features, matching the proposed model’s configuration.

The FC model consists of three fully connected layers with ReLU activations, using dropout for regularization. It takes flattened input sequences and processes them through progressively reduced hidden layers, consistent with prior studies [34,45,46]. Another widely adopted approach, the 2D CNN + FC model [47,48], was also implemented and evaluated under the same experimental conditions. This model treats the input as a 2D matrix (sequence length × sensors) and applies stacked convolutional layers with 64 and 128 filters, followed by adaptive pooling and a three-layer fully connected classifier.

Recognizing that each sensor stream has distinct temporal characteristics, a 1D CNN + FC model shown to be effective in PHM applications [23,30,49] was included in the comparison. This model uses a dedicated convolutional path for each sensor stream, applying two 1D convolutional layers (with two and four filters, respectively), followed by max pooling and two fully connected layers for classification. Lastly, to capture the sequential dependencies inherent in sensor data, LSTM + FC models were evaluated [50,51,52]. The model uses a two-layer bidirectional LSTM with a hidden size of 64. Its final hidden state is passed through two fully connected layers for final classification.

For all models, we used a batch size of 64, a learning rate of 0.001, and the Adam optimizer, consistent with the proposed model. Training was carried out until the validation loss plateaued or began to decline, and the best-performing checkpoint was selected for final evaluation. These baseline models were selected to represent a range of widely adopted deep learning strategies in PHM, including feedforward, convolutional, and sequence-based architectures.

**Figure 8:**
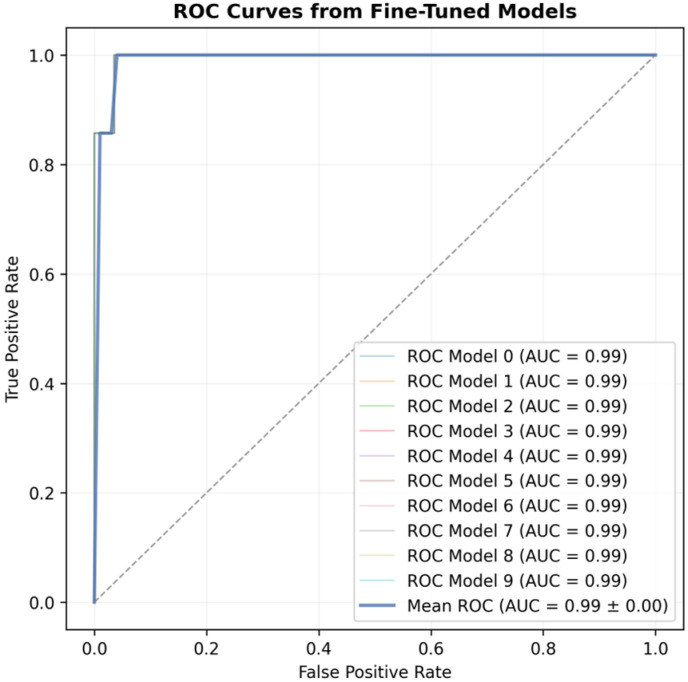
ROC curve of fine-tuned models in the K-fold cross-validation with 10 folds. Dashed line represents a random classifier.

Figure 8 illustrates the Receiver Operating Characteristic (ROC) curves for the fine-tuned models across all folds of the K-fold cross-validation. The Area Under the Curve (AUC) consistently reaches 0.99 for all folds, indicating strong model performance and suggesting a uniform distribution across the dataset partitions. Furthermore, the presence of sharp transitions and jagged edges in the ROC curves reflects the class imbalance, characterized by a significantly higher number of healthy data points.

### 5.3. Ablation Study

**Figure 9:**
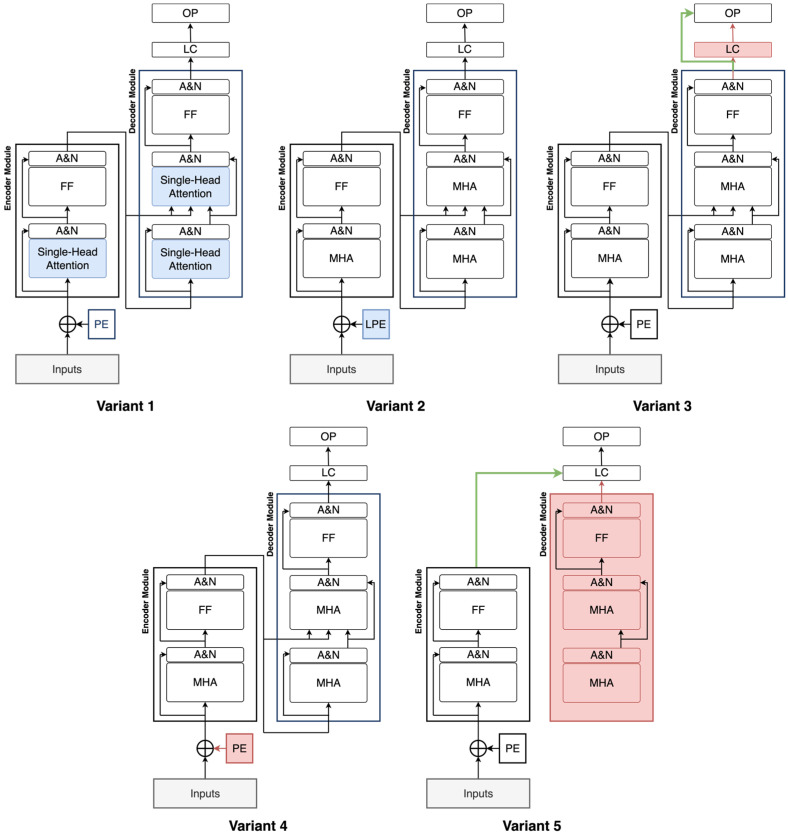
Ablation study variants. Blue: altered entity, red: removed entity, and green: added entity. A and N: add and normalization layer, FF: feedforward network, PE: positional encoding, LC: linear classifier layer, OP: output probabilities, MHA: multi-head attention, LPE: learnable positional encoding.

To evaluate the contribution of each subsystem in the proposed model, an ablation study is conducted by systematically modifying or removing specific components. Each variant is pre-trained on the source dataset and subsequently fine-tuned on the target dataset using K-fold cross-validation, and the results are compared to those of the original model. Five model variants are created for this study as depicted in Figure 9:

- Variant 1: The multi-head attention layer is replaced with a single-head attention layer to assess the impact of using multiple attention heads.
- Variant 2: The sinusoidal positional encoding is substituted with a learnable positional encoding to evaluate the benefits of fixed versus trainable positional information.
- Variant 3: The hidden layer in the classifier is removed and replaced with a single fully connected (FC) layer to reduce complexity.
- Variant 4: The positional encoding is entirely removed to test the model’s reliance on positional information.
- Variant 5: The decoder module is eliminated, and the encoder is directly connected to the final classifier to isolate the encoder’s contribution.

Table 4 shows the ablation study results. As each subsystem is systematically removed or altered, the final performance is reduced, as shown in the table. This demonstrates the importance and complementary contribution of each component within the overall model architecture to its high performance.

### 5.4. SHAP Explainability Results

At the final stage, following model training and validation, it is essential in engineering applications to analyze the model’s output and quantify the contribution of each sensor input to the prediction. This step provides insight into the internal decision-making process of the model. Given that the model’s output directly influences maintenance-related decisions, it is crucial for system operators to not only verify the reliability of the prediction but also to identify the specific sensors and data points that contributed most significantly to the outcome. Such interpretability facilitates a more informed and rapid response to potential faults, thereby improving system recovery and enhancing operational reliability.

**Figure 10:**
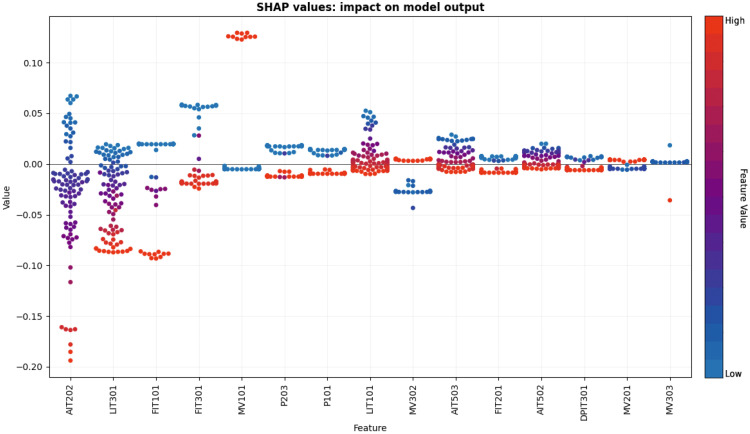
SHAP summary plot of the top 15 features influencing the model’s predictions. Each dot represents a test sample, where color indicates the feature value (red is higher), and the Y-axis shows its impact on predicting faulty or healthy states.

Figure 10 presents the top 15 features that contribute most significantly to the model’s output based on SHAP values. Each dot represents a single test sample, and the color indicates the actual normalized value of the corresponding feature: red for high values and blue for low values. The position of each dot along the Y-axis reflects the SHAP value, which quantifies the feature’s impact on the model’s prediction for that specific sample. Positive SHAP values indicate a contribution toward predicting the faulty class, while negative values contribute toward predicting the healthy class. The spread and density of the dots reveal how consistently each feature influences the prediction across the dataset. For instance, features such as AIT202 (sensor measuring the HCl level in the second stage) and LIT301 (feed water tank level in the third stage) show strong and consistent influence in one direction, suggesting they are critical indicators of the system’s health state. This plot shows the most influential features overall and provides detailed insight into how different values of each feature affect the model’s decision, which is essential for fault diagnosis and understanding the underlying behavior of the model.

**Figure 11:**
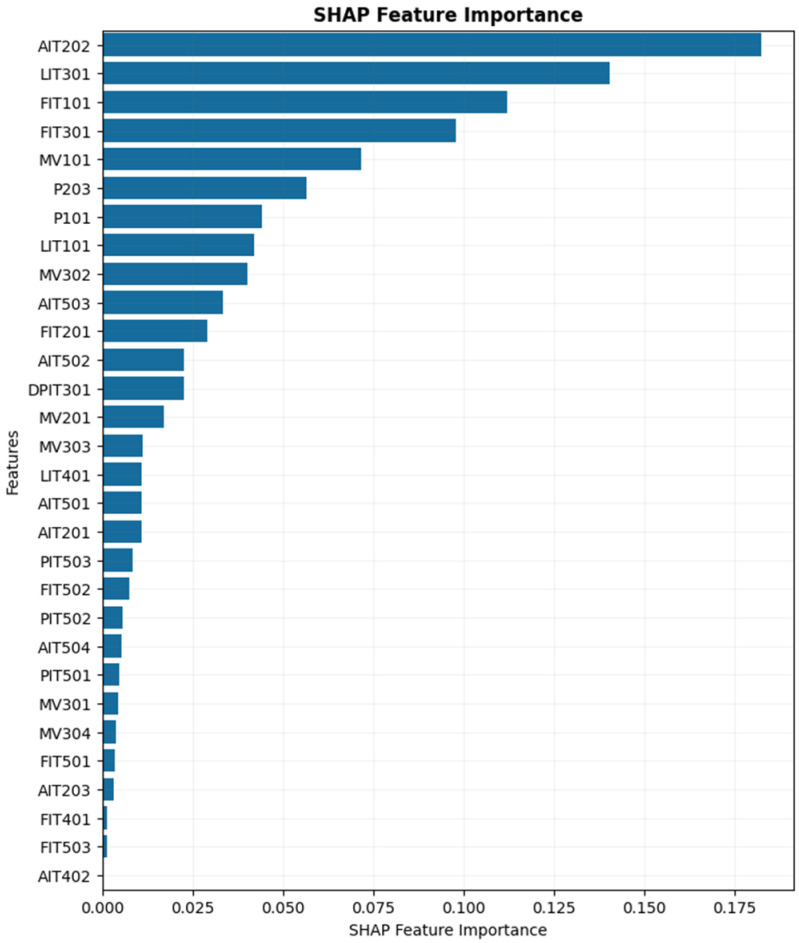
Global feature importance. Average of feature impact on model output.

Figure 10 shows the local interpretability for each individual prediction. To calculate the global feature importance, we can calculate the average on the absolute of SHAP values of all instances as per Equation (19). In this formula, $I_{j}$ stands for the global importance of feature j, n is the number of samples, ${\phi_{j}}^{\left(i\right)}$ is the SHAP value for feature j in sample i, and $\left|.\right|$ stands for the absolute operator. In Figure 11, features are sorted based on their global importance.

(19) $$I_{j}=\frac{1}{n}\sum_{i=1}^{n}\left|{\phi_{j}}^{\left(i\right)}\right|$$

As shown in Figure 11, the most influential features based on global SHAP values are AIT202, LIT301, FIT101 (flow meter sensor in the first stage), FIT301 (flow meter sensor in the third stage), and MV101 (motorized valve actuator in the first stage). These features exhibit the highest overall contribution to the model’s predictions, while the remaining features have comparatively minimal impact on the output.

### 5.5. Discussions

The experimental results demonstrate that the proposed pre-trained transformer methodology significantly improves classification performance on the SWaT.A6 dataset, particularly in terms of the F1-score for the attack class. While conventional models such as FC, CNN, and LSTM have previously shown reasonable effectiveness in PHM tasks, the incorporation of transfer learning from a larger dataset yielded a substantial performance gain in this study, achieving an F1-score of 93.38%. This improvement is primarily attributed to the effective transfer of temporal knowledge across operational domains. Pre-training on the SWaT.A1 dataset enables the model to learn generalized temporal features that remain relevant when fine-tuned on the smaller SWaT.A6 dataset. Notably, freezing the transformer layers during fine-tuning resulted in a considerable performance drop (F1-score decreased from 93.38% to 83.25%), highlighting the importance of continued adaptation. Additionally, our ablation study showed that transfer learning improved the F1-score by 8 percentage points compared to training from scratch, further emphasizing the critical role of pre-training in achieving high performance under data-constrained conditions.

The adoption of a simplified transformer architecture, consisting of a single encoder and decoder block, also contributed to reducing the risk of overfitting—a crucial concern when dealing with small-scale industrial datasets. This makes the proposed approach more practical for real-world industrial applications, where collecting and labeling large amounts of fault data is often expensive and time-consuming. Instead, models can be pre-trained on data collected under controlled laboratory conditions and subsequently adapted to specific operational environments with limited labeled data. However, despite the strong performance on the SWaT datasets, it is important to acknowledge the limitations. Both the source and target datasets are derived from the same CPS testbed, albeit recorded under different conditions and four years apart. The extent to which this methodology generalizes to entirely different CPS environments remains an open question. Future research should apply this approach to other domains, such as smart grids, autonomous systems, or manufacturing environments, to validate its broader applicability. Moreover, while a simplified transformer design was employed, transformer models inherently require more computational resources than CNNs or LSTMs. This could present deployment challenges in resource-constrained environments. Therefore, future work should consider incorporating model compression techniques and hardware-aware optimizations to improve deployment feasibility without sacrificing performance.

## 6. Conclusions and Future Directions

This study proposed a transfer learning approach using a pre-trained transformer model for fault detection in cyber-physical systems. The method was evaluated on the SWaT.A1 and SWaT.A6 datasets, achieving superior performance over traditional deep learning models, with an accuracy of 99.02% and an F1-score of 93.38%. These results highlight the effectiveness of pre-training on a larger dataset to extract transferable temporal features that remain useful when fine-tuned on a smaller target dataset under different operational conditions. This helps address a common challenge in industrial environments, where collecting and labeling large volumes of fault data is often impractical due to cost, time, and operational constraints.

Furthermore, to assess the interpretability of the proposed model, we applied explainable AI techniques using SHAP. This allowed us to analyze the contribution of individual sensor features to each prediction and identify the key factors influencing the model’s decision, thereby improving transparency and aiding fault diagnosis in CPS applications.

Future work will expand this methodology in three key directions. First, model interpretability will be enhanced by incorporating explainable AI techniques, enabling greater transparency in the decision-making process—an essential requirement for industrial adoption. Second, model compression strategies such as pruning and quantization will be explored to reduce computational overhead and inference latency, facilitating deployment on resource-constrained systems. Third, the approach will be validated on additional CPS datasets with varying configurations and fault scenarios to assess its robustness and generalizability across diverse industrial settings.

## Figures and Tables

**Table 1:** The proposed methodology details.

Methodology Section	Component	Description
Transformer Module	Model dimension	64
	Encoder count	1
	Input dimension	30
	Number of heads	4
	Decoder count	1
	Position embedding	Sin Cos
Classifier Module	Layer shapes	30 to 16 to 64 to 2
	Activation function	ReLu
Process Parameters	Window size	16
	Batch size	64
	Learning rate	0.001

**Table 2:** Proposed methodology comparison with state-of-the-art.

Methodology	Accuracy	Precision	Recall	F1-Score
Bahadoripour et al. [37]	99.6	99.6	99.6	99.6
Jahromi et al. [40]	95.1	95.3	95.1	95.2
Jahromi et al. [41]	90.83	90.98	90.83	90.90
Bahadoripour et al. [42]	99.0	99.0	98.0	98.0
Nedeljkovic and Jakovljevic [43]	97.9	98.8	83.0	90.2
Moon et al. [44]	-	86.51	87.82	87.16
Our proposed method	99.79	98.95	99.39	99.17

**Table 3:** Performance of the fine-tuned model on the target dataset compared to state-of-the-art approaches.

Methodology	Accuracy	F1-Score	*p*-Value
Locked Transformer Weights	97.50 ± 0.50	83.25 ± 4.18	5.00 × 10^−6^
Model Without Transfer Learning	97.71 ± 0.90	85.20 ± 5.63	7.59 × 10^−4^
FC	96.95 ± 0.81	79.61 ± 6.92	1.45 × 10^−4^
2D CNN + FC	96.74 ± 0.68	72.78 ± 8.13	1.01 × 10^−5^
1D CNN + FC	97.61 ± 0.81	84.74 ± 4.56	5.26 × 10^−4^
LSTM + FC	96.95 ± 0.81	78.64 ± 5.67	1.53 × 10^−5^
Our proposed method	**99.02 ± 0.33**	**93.38 ± 2.25**	-

**Table 4:** Performance of the ablation variants.

Methodology	Accuracy	F1-Score
Variant 1	97.55 ± 0.90	83.99 ± 6.24
Variant 2	97.82 ± 0.76	85.93 ± 4.35
Variant 3	98.09 ± 1.18	88.30 ± 7.29
Variant 4	98.37 ± 0.54	89.26 ± 3.57
Variant 5	98.09 ± 0.47	86.17 ± 3.57
Our proposed method	**99.02 ± 0.33**	**93.38 ± 2.25**

## Data Availability

SWaT dataset used in the publication is publicly available. All codes and software used for this paper are open source and available at https://github.com/pooyasa/pre-trained-trasformer-fd (accessed on 12 June 2025).

## Abbreviations

The following abbreviations are used in this manuscript:

AUC	Area Under the Curve
BF	Brute Force
CNNs	Convolutional Neural Networks
CPS	Cyber-Physical Systems
CWT	Continuous Wavelet Transform
DAST	Dual-Aspect Self-Attention Transformer
DDoS	Distributed Denial of Service
DL	Deep Learning
Dos	Denial of Service
ELM	Extreme Learning Machine
FC	Fully Connected
IOTs	Internet of Things
K	Key
KNNs	K-Nearest Neighbors
LSTM	Long Short-Term Memory
MB	Mirai Botnet
ML	Machine Learning
MMD	Maximum Mean Discrepancy
NLP	Natural Language Processing
PCA	Principle Component Analysis
PHM	Directory of open access journals
PLCs	Programmable Logic Controllers
PMU	Phasor Measurement Unit
Q	Query
ReLU	Rectified Linear Unit
ResNet	Residual Network
RNNs	Recurrent Neural Networks
ROC	Receiver Operating Characteristic
RUL	Remaining Useful Life
SAE	Stacked Auto-Encoder
SCADA	Supervisory Control and Data Acquisition
SD	Source Dataset
SHAP	Shapley Additive Explanations
SMOTE	Synthetic Minority Over-sampling Technique
SVR	Support Vector Machine
SWaT	Secure Water Treatment
TD	Target Dataset
V	Value
XAI	Explainable Artificial Intelligence
